# Supplementary material for: SARS-CoV-2 infection severity and mortality is modulated by repeat-mediated regulation of alternative splicing
Source: Microbiol Spectr. 2023 Aug 21;11(5):e01351-23. doi: 10.1128/spectrum.01351-23 (PMC10580830; doi:10.1128/spectrum.01351-23)
Supplement: Figure S1 — Clinical features and transcript diversity characterization across moderate and severe patients. [file spectrum.01351-23-s0001.pdf]

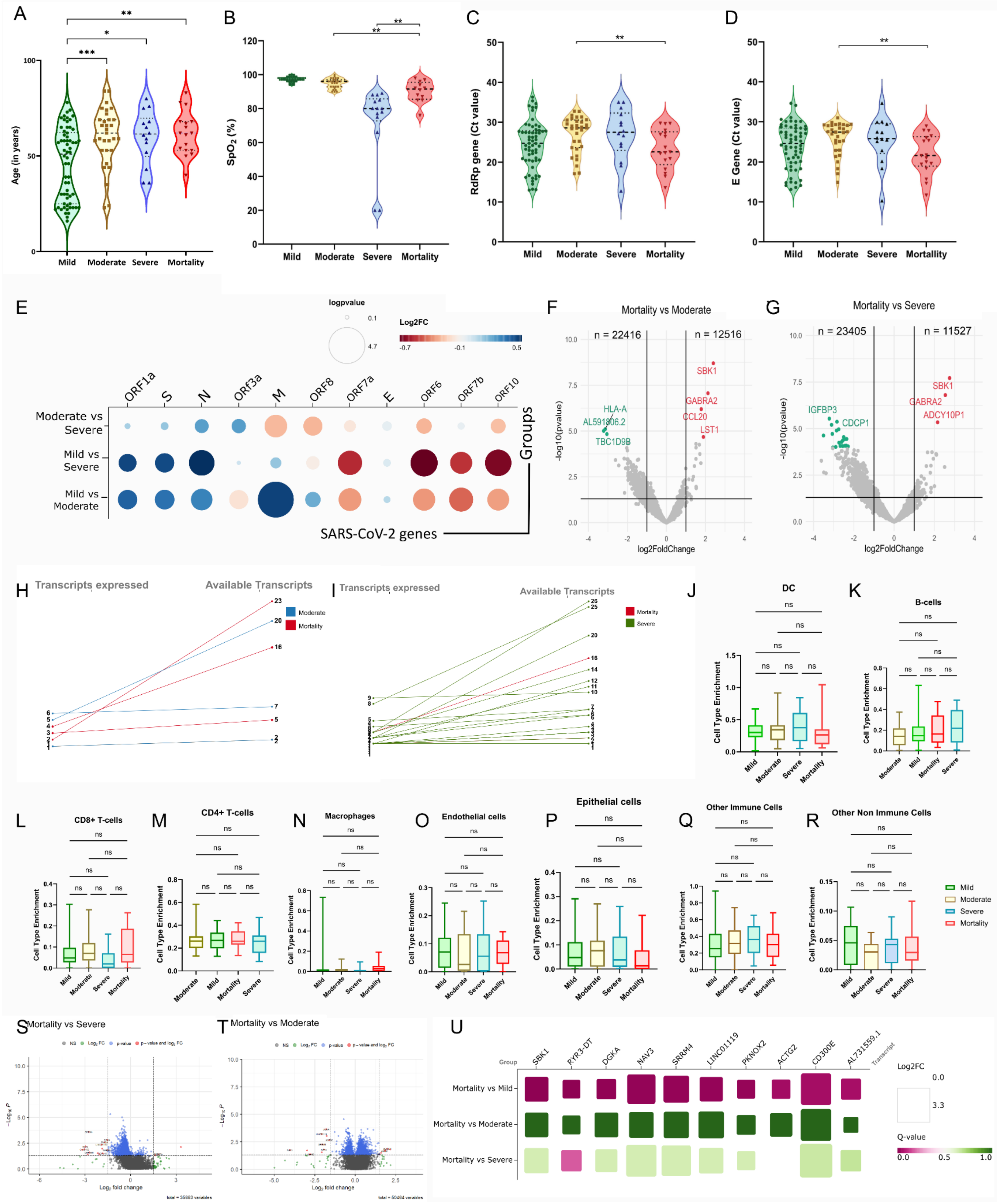

Supplementary Figure 1: Clinical features and transcript diversity characterisation across severity groups. The violin plot represents comparison of **A)** Age, **B)** SpO_2, **C)** RdRp and **D)** E gene across mild, moderate, severe and mortality. **E)** Differential gene expression (DEG) of SARS-CoV-2 genes between mild moderate and severe groups. The volcano plot represents the DEGs between **F)** Moderate vs Mortality and **G)** Severe vs Mortality groups. The slope plot represents the transcripts expressed (left) and available transcripts (right) for DEGs in **H)** Mortality and Moderate, **I)** Mortality and Severe groups. **J-R)** Distribution of different cell types based on Xcell analysis in subgroup. The differentially expressed transcripts in clinical subgroups. Violin plot of differential transcript expression between **S)** Mortality vs Moderate, **T)** Mortality vs Severe groups. **K)** Expression pattern of 10 significantly expressed transcripts in Mortality vs Mild group in other comparison groups (namely Mortality vs Moderate/Severe). All log fold changes in volcano plots are based on second group in header.
