# Supplementary material for: SARS-CoV-2 infection severity and mortality is modulated by repeat-mediated regulation of alternative splicing
Source: Microbiol Spectr. 2023 Aug 21;11(5):e01351-23. doi: 10.1128/spectrum.01351-23 (PMC10580830; doi:10.1128/spectrum.01351-23)
Supplement: Figure S2 — Splicing pattern and repeat element distribution in the differentially expressed transcripts. [file spectrum.01351-23-s0002.pdf]

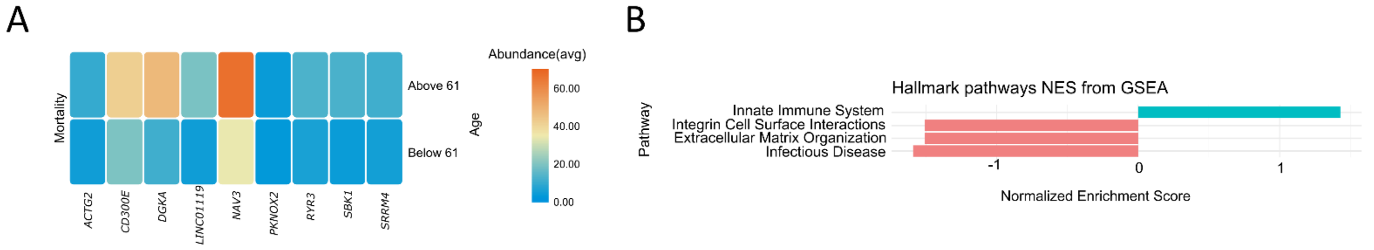

C

Retained intron genes	DTE(Sig) n=19	DI (Non Sig) n=62	P-value
SE	2.1	16.9	0.001
Alt 5'	14.6	15.8	0.36
Alt 3'	8.3	7.3	0.9
RI	14.6	14.2	0.10
Alt P	31.3	23.5	**0.29**
Alt T	29.2	23.1	0.60

D

Processed transcripts	DTE(Sig) n=10	DI (Non Sig) n=21	P-value
SE	13.6	17.89	0.44
Alt 5'	13.6	8.94	0.51
Alt 3'	9.1	8.94	0.94
RI	13.6	14.63	0.7
Alt P	27.3	26.02	0.94
Alt T	22.7	23.58	0.71

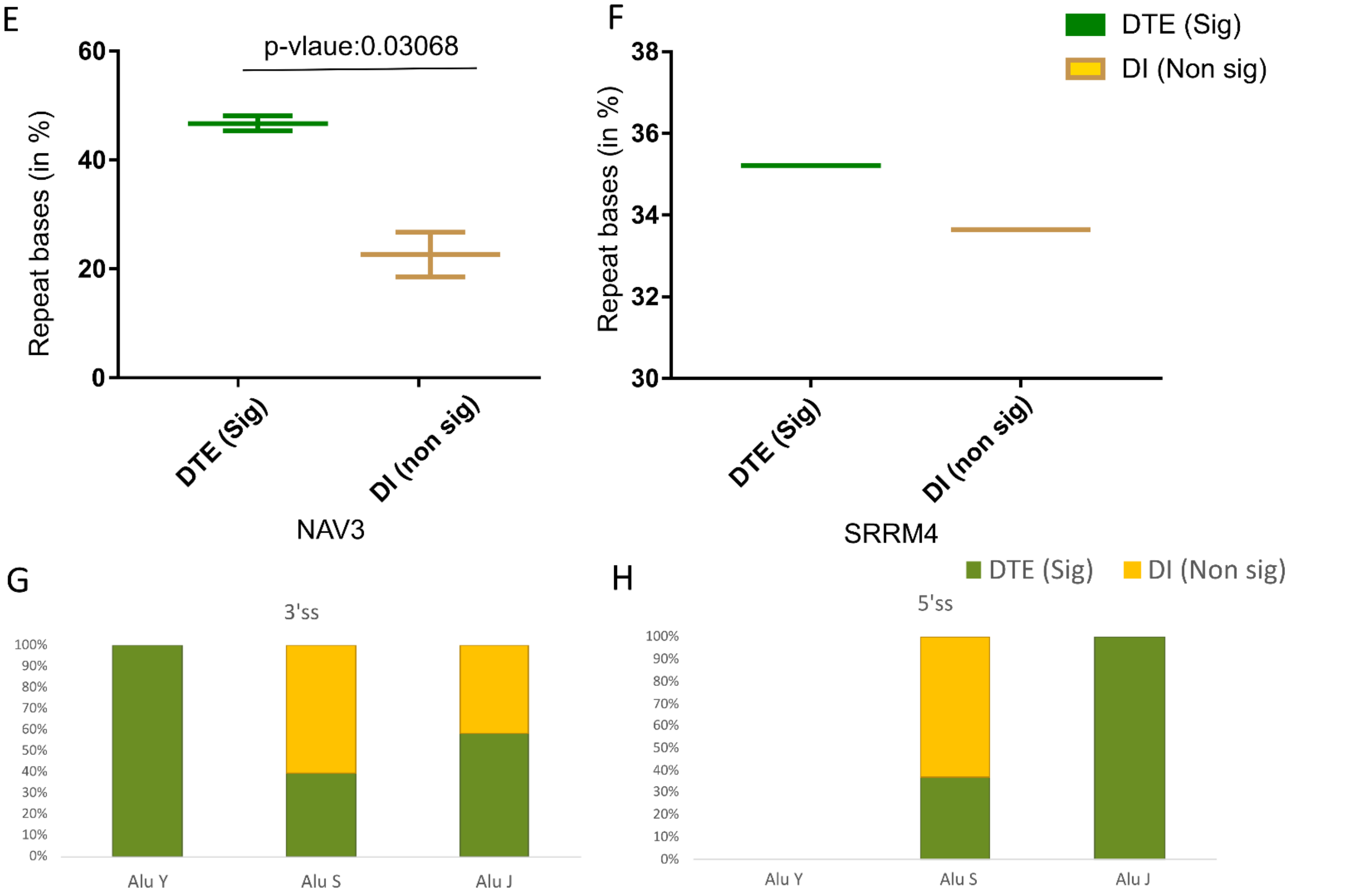

Supplementary Figure 2: Splicing pattern and repeat element distribution in differentially expressed transcripts. A) Average abundance distribution of mortality patients divided based on their median age. Patients above 61 years of age and patients below 61 years of age. It does not include the pseudogene, AL731559.1. B) Pathway enrichment analysis using reactome for 121 differentially expressed transcripts. Splicing pattern across in different biotypes: C) retained intron and D) processed transcript. Total repeat bases comparison in the promoter region of DTE and DI for E) NAV3 and F) SRRM4 proteins. Distribution of Alu subfamilies in DTE and DI at the G) 3’ splice site and H) 5’ splice sites. Green represents DTEs and DI is represented in yellow.
